# Supplementary material for: The genome sequence of the American mink, Neogale vison (Schreber, 1777)
Source: Wellcome Open Res. 2025 Sep 26;10:103. Originally published 2025 Feb 24. [Version 2] doi: 10.12688/wellcomeopenres.23780.2 (PMC12519072; doi:10.12688/wellcomeopenres.23780.2)
Supplement: Peer review history

# Reviewer report: Reviewer response for version 2

**DOI:** 10.21956/wellcomeopenres.27551.r134680

**Published:** 2025-10-13

**Authors:** Manuel Ruedi

**Affiliations:** 1 Université de Genève, Genève, Switzerland

The current version of the ms is excellent and clear, and I have only the following few changes to propose:

*Arvicola terrestris* is now considered a junior synonym of *Arvicola amphibius* (refer 1). How many mammal species are there now? Updates and trends in taxonomic, nomenclatural, and geographic knowledge. *Journal of Mammalogy* in press: TBD.).

 “and intraspecific aggression” should be replaced by “and interspecific aggression” ( *Neovison vison* versus *Mustela lutreola*!)

 I would suggest that it is “native to North America but introduced elsewhere”.

 Perhaps repeat in the legend of the picture of dead mink that this specimen was a female, as this, of course, will immediately explain the absence of Y chromosome in its genome.

Are sufficient details of methods and materials provided to allow replication by others?

Partly

Is the rationale for creating the dataset(s) clearly described?

Yes

Are the datasets clearly presented in a useable and accessible format?

Yes

Are the protocols appropriate and is the work technically sound?

Yes

Reviewer Expertise:

Taxonomy mammalogy systematics

I confirm that I have read this submission and believe that I have an appropriate level of expertise to confirm that it is of an acceptable scientific standard.

## References

[1] : How many mammal species are there now? Updates and trends in taxonomic, nomenclatural, and geographic knowledge. *bioRxiv* .2025; 10.1101/2025.02.27.640393 10.1101/2025.02.27.640393 PMC1252694141103548

# Reviewer report: Reviewer response for version 2

**DOI:** 10.21956/wellcomeopenres.27551.r134425

**Published:** 2025-10-13

**Authors:** Richard Edwards

**Affiliations:** 1 University of Western Australia, Perth, Australia

I don’t think the authors have responded to my review. They appear to have addressed my minor comments but have failed to address the question of why the species was sequenced again. I requested a basic comparison with the previous assembly, which still appears to be absent. As a result, my Approval Status of "Approved with Reservations” still stands.

Are sufficient details of methods and materials provided to allow replication by others?

Partly

Is the rationale for creating the dataset(s) clearly described?

Partly

Are the datasets clearly presented in a useable and accessible format?

Yes

Are the protocols appropriate and is the work technically sound?

Yes

Reviewer Expertise:

NA

I confirm that I have read this submission and believe that I have an appropriate level of expertise to confirm that it is of an acceptable scientific standard, however I have significant reservations, as outlined above.

# Reviewer report: Reviewer response for version 1

**DOI:** 10.21956/wellcomeopenres.26228.r119476

**Published:** 2025-06-23

**Authors:** Ellie E Armstrong

**Affiliations:** 1 Evolution, Ecology, and Organismal Biology, University of California Riverside (Ringgold ID: 8790), Riverside, California, USA

The report from O’Brien and Lopez Colom presents a novel, highly contiguous American mink genome from a female specimen collected in the U.K. The report is mostly complete and contains a fairly complete description of methods (though some of these are unpublished and have paper in progress placeholders), but the introduction of the manuscript would benefit from a more structured and cohesive presentation. At present, it appears somewhat abrupt and may not effectively guide the reader into the study's context and objectives. Enhancing the flow and clarity of this section could provide a clearer foundation for the research presented. I also find it surprising that the report does not make a direct comparison to the previous American mink genome in the publication, which is fairly standard even for genome reports. I offer some brief, line by line suggestions for improvement below.

 ‘is a semi-aquatic mustelid which’ change ‘which’ to ‘that’

 ‘with white patches at chin’ change to ‘at the chin’

 ‘in the wild, males’ change to ‘in the wild, with males weighing up to’

 The MaCdonald in ‘MacDonald and Harrington’ has incorrect capitalization?

 Sentence about decline awkward with in the UK and in Europe, rephrase ‘They have been major contributors to the decline of the water vole in the UK and the European mink in Europe, due to predation…’

 ‘The range of the American mink’ this seems out of place, perhaps move this up to the first paragraph?

 ‘Study into the genome of individuals in the UK may therefore feed’ rephrase, awkward

 ‘functional genetic diversity’ what is the functional part of this sentence implying?

 The samples for RNA and DNA seem like they are from different the same sample, but the HiC is from a different sample based on Table 1, but in the methods it seems like these are all from the same individual (mNeoVis2)?

 Might want to add which BUSCO lineages were used. Was the RNA data used at all or just generated?

 In Table 2 I am surprised you are calling this span instead of just total length or something more standard?

 I think it would be useful to add in stats comparing the previous assembly from 2022.

Are sufficient details of methods and materials provided to allow replication by others?

Partly

Is the rationale for creating the dataset(s) clearly described?

Yes

Are the datasets clearly presented in a useable and accessible format?

Yes

Are the protocols appropriate and is the work technically sound?

Yes

Reviewer Expertise:

Genome biology, conservation genomics, population genetics

I confirm that I have read this submission and believe that I have an appropriate level of expertise to confirm that it is of an acceptable scientific standard, however I have significant reservations, as outlined above.

# Response

**Published:** 2025-09-21

**Authors:** Tree of Life Team Sanger

**Affiliations:** Wellcome Trust Sanger Institute, UK

Thank you for reviewing this data note. Here are some of our responses and points of clarification:

- We have edited the Background section to enhance flow and clarity as you suggest.
- We have changed the capitalisation of “MacDonald and Harrington”, but note the capitalisation in the original reference: https://www.tandfonline.com/doi/abs/10.1080/03014223.2003.9518350.
- Samples for RNA and DNA: all samples were taken from the same individual (mNeoVis2). The BioSample accessions are linked to the submitted tubes, and different BioSample accessions indicate that a different tube of tissue was used for Hi-C sample preparation.
- We clearly state in the footnote to Table 1, in Figure 2 and in the methods section that the BUSCO lineage used for analysis was carnivora_odb10.
- The RNA data is generated and released under the same BioProject, but is not used in the assembly steps. The genome has been annotated by the European Bioinformatics Institute since version 1 of the data note, and we have included this information in version 2.
- We commonly use the word “span” rather than “length” in data notes to avoid the implication that the sequence is physically continuous. We have changed it to “Length” in Table 2.
- Thank you for the suggestion to compare the statistics of the previous chromosome-level genome sequence of *Neogale vison* ( https://www.ncbi.nlm.nih.gov/datasets/genome/GCF_020171115.1/). We have chosen not to include a direct, side-by-side comparison of assembly statistics because the two genomes differ in specimen origin (North America vs UK), biological background (strain/population), and methods/pipelines. Our aim in a data note is to provide a high-quality genomic resource with clear provenance and methods so that researchers can make the comparisons most relevant to their questions. Both assemblies are chromosome-level with high completeness. Interested readers can readily conduct formal comparisons using the deposited data and methods we provide.

# Reviewer report: Reviewer response for version 1

**DOI:** 10.21956/wellcomeopenres.26228.r119471

**Published:** 2025-04-05

**Authors:** Gabriela Pozo

**Affiliations:** 1 Universidad San Francisco de Quito, Quito, Ecuador

The article is a very clear description of the high-quality genome assembly of the American mink.

**Comments:**

 1. In the background section, I believe it would be helpful to combine paragraphs so that they are not on or two sentences. For example, I would combine paragraphs 1 and 2, as well as 5 and 6.

 2. The last paragraph mentions that the genome has been sequenced previously. There is no justification or explanation as to why it is being re-sequenced. What would this genome be used for. I believe this would strengthen the article.

 3. In the methods section I suggest providing more detail regarding:

 - RNA sequencing parameters

 - Assembly - I suggest providing more details regarding the assembly and mapping (what parameters were used). This comment applies to both the nuclear and mitochondrial genome.

Are sufficient details of methods and materials provided to allow replication by others?

Partly

Is the rationale for creating the dataset(s) clearly described?

Partly

Are the datasets clearly presented in a useable and accessible format?

Yes

Are the protocols appropriate and is the work technically sound?

Yes

Reviewer Expertise:

Genomics

I confirm that I have read this submission and believe that I have an appropriate level of expertise to confirm that it is of an acceptable scientific standard, however I have significant reservations, as outlined above.

# Response

**Published:** 2025-09-21

**Authors:** Tree of Life Team Sanger

**Affiliations:** Wellcome Trust Sanger Institute, UK

Thank you for reviewing this data note. We have submitted a new version of the genome note and we respond to each of your concerns below.

**Reviewer comment 1.** In the background section, I believe it would be helpful to combine paragraphs so that they are not on or two sentences. For example, I would combine paragraphs 1 and 2, as well as 5 and 6.

*Response: We have combined paragraphs and edited the Background section.*  

**Reviewer comment 2.** The last paragraph mentions that the genome has been sequenced previously. There is no justification or explanation as to why it is being re-sequenced. What would this genome be used for. I believe this would strengthen the article.

*Response: We have added information on the rationale of sequencing a UK mink specimen.*

**Reviewer comment 3.** In the methods section I suggest providing more detail regarding: - RNA sequencing parameters - Assembly - I suggest providing more details regarding the assembly and mapping (what parameters were used). This comment applies to both the nuclear and mitochondrial genome.

*Response: We have added more detail to the RNA library preparation and sequencing section in Version 2. The full details of assembly are given in the section “Genome assembly, curation and evaluation”. In addition, we have now linked to the pipelines used for genome assembly (* https://github.com/sanger-tol) in the “Data availability” section.

# Reviewer report: Reviewer response for version 1

**DOI:** 10.21956/wellcomeopenres.26228.r119469

**Published:** 2025-03-18

**Authors:** Richard Edwards

**Affiliations:** 1 University of Western Australia, Perth, Australia

This manuscript describes a high quality phased genome assembly for an important UK invasive species, the American mink. The assembly data and methods are generally well described and are of high quality. However, as the authors point out at the end of the Introduction, this species already has a reference genome - itself assembled to chromosome-level. This raises the question as to why the species was sequenced again. Are there important population differences between the Canadian mink and the UK mink? Or were there issues identified with the original genome that this new assembly sought to rectify? A second female was selected for sequencing, so it was not to add the Y chromosome. Was it to get a phased version? (The original appears to be a primary haploid assembly.) 

 Whilst I appreciate that this is a genome report with limited analysis, I think it is important to present the statistics of the original genome assembly (both genome stats and chromosome sizes) alongside those of the new assembly. This is important to help guide the reader concerning which assembly they should use for different purposes.

**Minor Comments:**

- Typo - rogue capitalisation: MaCdonald DW, Harrington LA: The American mink: the triumph and tragedy of adaptation out of context. N Z J Zool. 2003; 30(4): 421–441. 
- Curation: At two points, details are missing and cited as “(article in preparation).” This needs to be described or a link provided to published details, e.g. a pre-print or similar, or a personal communication from named authors. The rapid curation GitHub citation needs to link to a specific version/release or, if this is not possible (there do not appear to be versions or releases), provide the date of access and the github commit hash.

Are sufficient details of methods and materials provided to allow replication by others?

Partly

Is the rationale for creating the dataset(s) clearly described?

Partly

Are the datasets clearly presented in a useable and accessible format?

Yes

Are the protocols appropriate and is the work technically sound?

Yes

Reviewer Expertise:

Biodiversity genomics, genome assembly, bioinformatics, molecular evolution.

I confirm that I have read this submission and believe that I have an appropriate level of expertise to confirm that it is of an acceptable scientific standard, however I have significant reservations, as outlined above.
